# Personalised Nutrition: Updates, Gaps and Next Steps

**DOI:** 10.3390/nu11081793

**Published:** 2019-08-02

**Authors:** Jessica R. Biesiekierski, Katherine M. Livingstone, George Moschonis

**Affiliations:** 1 Department of Dietetics, Nutrition and Sport, School of Allied Health, Human Services and Sport, La Trobe University, Bundoora VIC 3086, Australia; 2 Institute for Physical Activity and Nutrition (IPAN), School of Exercise and Nutrition Sciences, Deakin University, Geelong VIC 3125, Australia

## 1. Introduction

Personalised nutrition approaches provide healthy eating advice tailored to the nutritional needs of the individual. Although there is no one definition for personalised nutrition, advice has typically been based on the individual’s behaviours, biological characteristics, and their interactions [1]. The objective of personalised nutrition is to improve dietary habits for the prevention or treatment of chronic disease, ultimately contributing to improvements in public health [2].

Two levels of the personalisation of nutrition advice have been conceived, which are based on the analysis of current behaviours, phenotypic characteristics and biological responses to diet [3]. The first level of personalised nutrition incorporates current behaviours and phenotypic characteristics (such as adiposity) to develop tailor-made dietary recommendations. The second level of personalised nutrition builds on the first layer but also takes into consideration the different responses to foods and/or nutrients that are dependent on genotypic or other biological characteristics [3]. 

Although there is some randomised controlled trial (RCT) evidence for the effectiveness of personalised nutrition advice [4], the scientific basis for personalisation of dietary advice is still in its infancy. The studies in this special issue of “Nutrients” bring together a series of recent clinical trials and review articles that present new data and update critical thinking to the current scientific basis that underpins personalised nutrition. 

### 1.1. Behavioural Level of Personalised Nutrition

The first level of personalisation of nutrition advice requires the collection of information on an individual’s current eating habits, behaviours and phenotypic characteristics [3]. These data are combined to provide personalised dietary advice tailored to these characteristics.

Maintaining sustained behavioural changes in personalised nutrition interventions is critical. Recent advances in technology have led to the development of behavioural tools to better facilitate adherence to personalised nutrition interventions. An example of this is demonstrated by Moschonis et al., who developed a computerised decision-support tool (DST) for use by paediatric healthcare professionals. The authors conducted an RCT designed to provide appropriate personalised nutrition meal plans and lifestyle recommendations in 35 overweight children and their families with healthcare professional support [5]. After three months of intervention, the group receiving advice through the DST showed improved changes in dietary patterns and body weight composition compared to the control group that received general recommendations [5].

### 1.2. Biological Levels of Personalised Nutrition

A number of studies in this special issue contributed to the scientific basis for personalisation based on biological characteristics (i.e., biomarkers, genotype, and microbiota). This includes understanding of the biological response due to dietary modifications, ranging from high-carbohydrate or high-fat meal challenges to whole diet interventions, and indicators of health and disease risk, including diabetes, obesity and appetite regulation.

#### 1.2.1. Biomarkers

Hjorth et al. utilised fasting plasma glucose, fasting insulin and a homeostatic model assessment of insulin resistance (HOMA-IR) as prognostic markers of long-term weight loss. These biomarkers were assessed in 811 overweight adults following diets differing in carbohydrate, fat, and protein content [6]. After 24 months of dietary intervention, subjects with normal glycemia lost the most weight on the low-fat/high-protein diet, subjects with high HOMA-IR had the highest weight loss on the high-fat/high protein diet, and subjects with prediabetes and low fasting insulin benefited most from higher intakes of dietary fibre (≥35 g/10 MJ) [6].

Glycemic control was also investigated by Kempf et al., who conducted a 12-week RCT in adults with type 2 diabetes risk (T2D) with poorly controlled glucose levels (HbA1c ≥ 7.5%). Individuals were randomised to either a two- or three-meal replacement therapy. In weeks 2–4 of the intervention, both groups reintroduced a low carbohydrate lunch based on individual adaption to self-monitoring of blood glucose (SMGB), followed by breakfast reintroduction after week four and a final follow-up period at week 12 [7]. The findings showed that the individualised meal replacement accompanied with SMBG demonstrated beneficial reduction in HbA1c and other cardiometabolic risk factors in T2D [7]. Furthermore, the initiation of such an approach led to clinically relevant long-term improvements in HbA1c, compared to an observational control group that had standard care [7].

Further insights into the effective design of personalised meal plans was reported in a study led by Adamska-Patruno et al. [8]. The authors conducted a crossover trial in 23 normal-weight and 23 overweight/obese adult males using meal challenges containing meals comprised of either a high-carbohydrate, normal carbohydrate or high-fat content [8]. Results showed that normal-weight men had higher adiponectin and lower total ghrelin response after the high-carbohydrate meal and the overweight/obese men showed higher fasting and postprandial leptin levels overall [8]. These findings demonstrate how differences in postprandial gastric hormone levels are dependent on macronutrient meal composition and baseline body weight [8], highlighting the importance of regulating satiation and appetite sensations in the design of personalised interventions.

#### 1.2.2. Genetics

Adamska-Patruno et al. conducted an acute meal-challenge study exploring gene variants and metabolites for T2D [9]. A total of 28 non-diabetic men were divided into either high risk or low risk according to carriage of the rs340874 SNP in the prospero-homeobox 1 (PROX1) gene [9]. A high or normal carbohydrate meal identified differences in postprandial metabolites associated with inflammatory and oxidative stress pathways, and bile acid signalling and lipid metabolism in PROX1 high-risk genotype men [9].

A systematic review performed by Brayner et al. evaluated the association between the FADS polymorphism, plasma long chain *n*-3 polyunsaturated fatty acids (PUFA) concentrations and risk of developing T2D [10]. Evaluation of five human observational studies and RCTs showed that FADS polymorphism may alter plasma fatty acid composition, therefore playing a protective role in the development of T2DM, while plasma *n*-3 PUFA levels were not associated with T2DM risk [10].

Taste receptor genes were investigated in an acute study in in 44 families to investigate taste function and dietary intake [11]. Chamoun et al. found key differences between children and parents as to which SNP in each of the sweet, fat, salt, umami and sour taste receptor genes was significantly associated with taste preference [11]. Furthermore, a multiple trait analysis of taste preference and nutrient composition of diet in the children revealed that rs9701796 in the TAS1R2 sweet taste receptor gene was associated with both sweet preference and percent energy from added sugar in the diet [11]. These findings suggest that for each taste preference, certain genetic variants are associated with taste function and thus, may be implicated in eating patterns.

#### 1.2.3. Microbiota

A review of gut microbiota composition as a prediction tool for the clinical response after dietary intervention was reported by Biesiekierski et al. [12]. Although there are data to show that the gut microbiota composition and inter-individuality in response to diet are linked, this review highlighted that current data are too limited and inconsistent to support specific microbial signatures predicting response to dietary interventions [12]. This was true for both weight loss and/or glycaemic response in obesity, and symptom improvement in irritable bowel syndrome.

## 2. Remaining Challenges and Future Steps

There are a number of remaining research questions that require elucidation before the implementation of personalised nutrition advice can be effectively and confidently incorporated into clinical practice. This special issue identified that the many factors responsible for inter-individual differences vary in response to diet and that there is a paucity of RCTs that incorporate all of these factors into the one personalised nutrition offering.

The existing literature and abovementioned studies show a predominate focus on weight management and markers for T2D and obesity. There remain many other disease cohorts that are yet to be explored in relation to the appropriateness of personalised nutrition approaches. One area is individualised allergen avoidance advice. D’Auria et al. contributed a review addressing this, and highlighted that although personalised nutritional management of IgE mediated food allergy has improved, especially with increased understanding of allergy phenotypes, more research is required [13]. 

To further assess genotype-based personalised nutrition, Drabsch and Holzapfel reported an overall lack of strong clinical evidence for using genetic variants for personalised dietary recommendations for weight management [14]. The authors highlighted the lack of evidence supporting the use of genetic direct-to-consumer tests by evaluating a number of commercial companies offering gene-based dietary recommendations for weight loss [14]. Multidisciplinary intervention studies are necessary to provide the appropriate evidence on the effectiveness of these commercial tests.

The findings presented in this special issue will help inform the development and implementation of personalised nutrition approaches. The suggested sequence for implementation should follow a step wise approach beginning with the simplest level of personalising dietary advice, based on dietary intake and behavioural and phenotypical characteristics, before progressing to the more complex level that includes the addition of biomarkers, genotypic and microbiota data [1]. Given the complexity of continually changing behavioural and biological information that are both influenced by diet and influence response to dietary interventions, the finer details of how best to implement such an approach are still to be elucidated through advances in big data and digital science. 

Future research to strengthen the evidence for personalised nutrition should include larger RCTs of longer intervention duration that aim to assess the effectiveness of personalised nutrition on long-term improvements in a variety of health outcomes. Moreover, future research should aim to address the current lack of consistency in the design of personalised advice across studies and their chosen methodologies [12]. This special issue will aid researchers in the design of more effective and comprehensive personalised nutrition research based on behavioural and biological characteristics.

## 3. Key Messages

This special issue on personalised nutrition presents a dynamic selection of reviews and original research in the ongoing development of evidence informing personalised nutrition strategies. Despite gaps in the scientific evidence, the future holds bright for the continued advancement of personalised nutrition, and ultimately how behavioural and biological characteristics can be integrated into step wise nutritional solutions specific to the needs of the individual for maintaining health and preventing disease. 

## References

[1] Celis-Morales C., Livingstone K.M., Marsaux C.F., Forster H., O’Donovan C.B., Woolhead C., Macready A.L., Fallaize R., Navas-Carretero S., Wim H.M. (2015). Design and baseline characteristics of the Food4Me study: A web-based randomised controlled trial of personalised nutrition in seven European countries. Genes Nutr..

[2] Gibney M., Walsh M., Goosens J., Eggersdorfer M., Kraemer K., Cordaro J.B., Fanzo J., Gibney M., Kennedy E., Labrique A., Steffen J. (2016). Personalized Nutrition: Paving the Way to Better Population Health, in Good Nutrition: Perspectives for the 21st Century.

[3] Ordovas J.M., Ferguson L.R., Tai E.S., Mathers J.C. (2018). Personalised nutrition and health. BMJ.

[4] Celis-Morales C., Livingstone K.M., Marsaux C.F., Macready A.L., Fallaize R., O’Donovan C.B., Woolhead C., Forster H., Walsh M.C., Navas-Carretero S. (2016). Effect of personalized nutrition on health-related behaviour change: Evidence from the Food4me European randomized controlled trial. Int. J. Epidemiol..

[5] Moschonis G., Michalopoulou M., Tsoutsoulopoulou K., Vlachopapadopoulou E., Michalacos S., Charmandari E., Chrousos G.P., Manios Y. (2019). Assessment of the Effectiveness of a Computerised Decision-Support Tool for Health Professionals for the Prevention and Treatment of Childhood Obesity. Results from a Randomised Controlled Trial. Nutrients.

[6] Hjorth M.F., Bray G.A., Zohar Y., Urban L., Miketinas D.C., Williamson D.A., Ryan D.H., Rood J., Champagne C.M., Sacks F.M. (2019). Pretreatment Fasting Glucose and Insulin as Determinants of Weight Loss on Diets Varying in Macronutrients and Dietary Fibers—The POUNDS LOST Study. Nutrients.

[7] Kempf K., Röhling M., Niedermeier K., Gärtner B., Martin S. (2018). Individualized Meal Replacement Therapy Improves Clinically Relevant Long-Term Glycemic Control in Poorly Controlled Type 2 Diabetes Patients. Nutrients.

[8] Adamska-Patruno E., Ostrowska L., Goscik J., Fiedorczuk J., Moroz M., Kretowski A., Gorska M. (2019). The Differences in Postprandial Serum Concentrations of Peptides That Regulate Satiety/Hunger and Metabolism after Various Meal Intake, in Men with Normal vs. Excessive BMI. Nutrients.

[9] Adamska-Patruno E., Godzien J., Ciborowski M., Samczuk P., Bauer W., Siewko K., Gorska M., Barbas C., Kretowski A. (2019). The Type 2 Diabetes Susceptibility PROX1 Gene Variants Are Associated with Postprandial Plasma Metabolites Profile in Non-Diabetic Men. Nutrients.

[10] Brayner B., Kaur G., Keske M., Livingstone K. (2018). FADS Polymorphism, Omega-3 Fatty Acids and Diabetes Risk: A Systematic Review. Nutrients.

[11] Chamoun E., Carroll N., Duizer L., Qi W., Feng Z., Darlington G., Duncan A., Haines J., Ma D. (2018). The Relationship between Single Nucleotide Polymorphisms in Taste Receptor Genes, Taste Function and Dietary Intake in Preschool-Aged Children and Adults in the Guelph Family Health Study. Nutrients.

[12] Biesiekierski J.R., Jalanka J., Staudacher H.M. (2019). Can Gut Microbiota Composition Predict Response to Dietary Treatments?. Nutrients.

[13] D’Auria E., Abrahams M., Zuccotti G., Venter C. (2019). Personalized Nutrition Approach in Food Allergy: Is It Prime Time Yet?. Nutrients.

[14] Drabsch T., Holzapfel C. (2019). A Scientific Perspective of Personalised Gene-Based Dietary Recommendations for Weight Management. Nutrients.

